# An Approach to Identify SNPs in the Gene Encoding Acetyl-CoA Acetyltransferase-2 (*ACAT*-*2*) and Their Proposed Role in Metabolic Processes in Pig

**DOI:** 10.1371/journal.pone.0102432

**Published:** 2014-07-22

**Authors:** Simrinder Singh Sodhi, Mrinmoy Ghosh, Ki Duk Song, Neelesh Sharma, Jeong Hyun Kim, Nam Eun Kim, Sung Jin Lee, Chul Woong Kang, Sung Jong Oh, Dong Kee Jeong

**Affiliations:** 1 Department of Animal Biotechnology, Faculty of Biotechnology, Jeju National University, Jeju-si, Jeju-do, South Korea; 2 The Animal Genomics and Breeding Center, Hankyong National University, Anseong-si, Gyeonggi-do, South Korea; 3 Department of Animal Biotechnology, College of Animal Bioscience and Technology, Kangwon National University, Chuncheon, South Korea; 4 Department of Mechanical and System Engineering, College of Engineering, Jeju National University, Jeju-si, Jeju-do, South Korea; 5 Sustainable Agriculture Research Institute (SARI), Jeju National University, Jeju-si, Jeju-do, South Korea; University of Westminster, United Kingdom

## Abstract

The novel liver protein acetyl-CoA acetyltransferase-2 (ACAT2) is involved in the beta-oxidation and lipid metabolism. Its comprehensive relative expression, *in silico* non-synonymous single nucleotide polymorphism (nsSNP) analysis, as well as its annotation in terms of metabolic process with another protein from the same family, namely, acetyl-CoA acyltransferase-2 (ACAA2) was performed in *Sus scrofa*. This investigation was conducted to understand the most important nsSNPs of ACAT2 in terms of their effects on metabolic activities and protein conformation. The two most deleterious mutations at residues 122 (I to V) and 281 (R to H) were found in ACAT2. Validation of expression of genes in the laboratory also supported the idea of differential expression of *ACAT2* and *ACAA2* conceived through the *in silico* analysis. Analysis of the relative expression of *ACAT2* and *ACAA2* in the liver tissue of Jeju native pig showed that the former expressed significantly higher (P<0.05). Overall, the computational prediction supported by wet laboratory analysis suggests that ACAT2 might contribute more to metabolic processes than ACAA2 in swine. Further associations of SNPs in ACAT2 with production traits might guide efforts to improve growth performance in Jeju native pigs.

## Introduction

The liver protein acetyl-CoA acyltransferase-2 (ACAA2), contributes to metabolism in two ways: it catalyses the last step of beta oxidation of mitochondrial fatty acids and is responsible for lipid catabolism. Apart from ACAA2, the ACAT2 protein is also expressed in the liver. The latter has two isoforms, ACAT1 and ACAT2, with distinct metabolic functions, intracellular localisations and membrane topologies in different mammals [1]–[2]. ACAT2 belongs to the same protein family as ACAA2 and both are present on chromosome 1 in pig.

Liver is one of the most important organs for metabolism and the partitioning of nutrients. It is involved in the transformation of dietary nutrients into fuel and their export via the blood. To meet the changing demands of extra-hepatic tissues for nutrients, liver displays remarkable metabolic flexibility [3]. The ACAT2 has a unique tissue distribution, being predominantly expressed in the liver and intestine [4]. It plays an important role in lipid and cholesterol metabolism in human beings and bovines. The high variability of ACAT2 was observed among the human liver samples and suggested that it is a regulated enzyme that is responsible for more than 50% of the ACAT activity in the majority of human population [4]. Another study, conducted using broiler chickens, investigated the expression of *ACAT2* in liver and revealed that this gene has been subjected to selection to either promote or suppress the accumulation of abdominal fat [5]. A correlation between *ACAT2* and cholesterol has also been reported in fish [6], and cholesterol has further been associated with increased lean mass in human beings [7]. Finally, ACAT2 controls enzymes involved in lipogenesis or lipolysis in beef cattle [8].

Moreover, analyses of single-nucleotide polymorphisms (SNPs) provide important information about genetic linkages, and are useful in fine-mapping of the regions of candidate genes. SNPs not only have attracted substantial attention in terms of human and vertebrate haplotypes associated with traits of interest, but they have also increased our understanding of the genetic basis of phenotypic diversity within and between populations [9]–[10]. Owing to their abundance in the genome, thousands of potentially informative SNP markers can be identified for the development of high-density SNP maps [11]. SNPs detected in the bovine ACAA2 are reported to be associated with gain in daily weight and loin muscle area (P<0.05) significantly [12]. Further, SNPs in the ovine *ACAA2* gene are reported to be associated with milk production traits [13]. Moreover, SNPs associated with production traits were discovered in the *ACAT2* gene of Holstein cattle [14].

Given the importance of the SNPs of *ACAT2* gene in metabolic processes, our study comprised two aspects. First, we conducted comprehensive *in silico* non-synonymous single nucleotide polymorphism (nsSNP) analysis and predicted the possible effects of these nsSNPs on protein structure and function. In the second stage, we explored the level of involvement of the *ACAT2* gene relative to that of *ACAA2* gene in metabolic processes in *Sus scrofa*.

## Materials and Methods

### Database retrieval of information on *ACAA2* and *ACAT2* genes

Data on the *ACAA2* and *ACAT2* genes from *Sus scrofa* and *Bos taurus* were collected from Entrez Gene of the National Centre for Biotechnology Information (NCBI). The SNP ids and types of SNPs related information were obtained from the NCBI dbSNP (http://www.ncbi.nlm.nih.gov/snp/) and SWISSProt databases (http://expasy.org/) for computational analysis. The online tool Treefam (http://www.treefam.org/) was used to analyze the phylogenic relationships between the *ACAA2* and *ACAT2* genes. A bootstrap threshold of 50% was used for the inclusion of organisms (bovine) in the same clade as Sus *scrofa.* It was followed by multiple sequence alignment to compare the sequences of the *ACAA2* and *ACAT2* from both bovine and swine.

### Annotation of Gene Ontology (GO) in terms of functional classes

GO analysis has become a commonly used approach for functional studies of large-scale genomic or transcriptomic data. In the current study, GO analysis has been used to analyse the biological processes associated with the functioning of the *ACAA2* and *ACAT2* genes in *S scrofa* and *B taurus*. CateGOrizer (previously known as “GO terms classifications Counter”; www.animalgenome.org/bioinfo/tools/countgo/) [15]; was used as a common language tool to annotate *ACAA2* and *ACAT2* in *S scrofa* and *B taurus*.

### Validation of the expression levels of *ACAA2* and *ACAT2* in *Sus scrofa* and *Bos taurus* liver tissues

#### Ethics Statement and collection of tissue samples

Pure bred adult animals from Jeju native pigs (JNPs) at an average body weight of 84.7±3.5 kg were used for the study. The study was carried out in strict accordance with the recommendations of the animal bioethics committee of Jeju National University, Jeju-Si, Jeju-Do, Republic of Korea. Bioethics committee specifically approved this study vide permit number: 2013-0009. Animals were reared under same environmental and nutritional conditions. The commercial feed (Seoul Feed, Jeju- Si, South Korea) and water were offered *ad- libitum* to all the pigs. Pigs were housed in pens with concrete flooring, a nipple bowl drinker and feeder. *Bos taurus* liver samples were collected from the Jeju joint livestock products market slaughter house, Jeju-Si, Republic of Korea. Five female animals of each species were used for the study and slaughtering method entailed exsanguination following electric stunning. The liver tissues were dissected immediately after slaughter and samples were frozen in liquid nitrogen. The tissue samples were subsequently stored in the laboratory at −80°C until the isolation of RNA and protein.

#### RNA and protein isolation from liver tissue

RNA was isolated from 100 mg of the fragmented frozen liver tissue samples of JNPs and bovines. *TRIzol* reagent (Invitrogen, USA) was used to isolate RNA. Tissue samples were homogenised in 1.5 ml of TRIzol reagent and chloroform, and the RNA extracted was subsequently precipitated using isopropanol (Junsei Chemical Co. Ltd., Japan). Isolated RNA samples were stored at −80°C. Genomic DNA contamination was removed by treating 25 µg of RNA from each sample with the RNase-free DNase set (QIAGEN, Hilden, Germany) and purification with the RNeasy mini kit, in accordance with the user guidelines (QIAGEN, Hilden, Germany). A Bioanalyzer 2100 with RNA 6000 Nano Labchips was used to assess the quality and quantity of RNA by automated capillary gel electrophoresis, in accordance with the user guidelines (Agilent Technologies, Dublin, Ireland). 28S/18S ratios for the RNA samples ranged from 1.8 to 2.0. Superscript III enzyme (Invitrogen, USA) was then used to synthesise first-strand cDNA.

Radio Immuno Precipitation Assay (RIPA) buffer was used to extract protein from the homogenised liver tissue. The manufacturer's guidelines were followed for measurement of the concentration of protein with the Pierce™ BCA protein assay kit (Thermo Scientific, USA) in a Bio-Rad Micro-plate Reader (Model-680). All the RNA and protein samples were stored at −80°C until use.

#### RT-PCR and quantitative real-time PCR

Primers used in RT-PCR and quantitative real-time PCR were designed using the online Primer-3 program [16] and the primers that were used to conduct PCR along with their detailed information are listed in Table 1. An Eppendorf Mastercycler gradient instrument was used to conduct RT-PCR under the following conditions: an initial 5 min at 94°C; 35 cycles of 30 seconds at 94°C, 30 seconds at the T_a_ temperature indicated in Table 1, and 1 min at 72°C; followed by a single extension period of 5 min at 72°C. The results of RT-PCR are presented as the relative expression normalised, using *GAPDH* transcript as an endogenous reference.

**Table 1:** List of primer sequences.

Gene name	Primer sequences	Product size	Annealing temperature (T_a_)	Genebank ID
Pig *ACAA2*	F- TAGGCTCTGTGGCTCTGGTT	225	61°C	NM_001167638.1
	R- GTAATTGCCATCGGGATTTG			
Bovine *ACAA2*	F- GTGTTCATCGTCGCTGCTAA	235	63.1°C	NM_001035342.2
	R- CTGGGGTCTCTTTTGGGATT			
Pig *ACAT2*	F- ATCACCAAGGAGCGAATCC	245	58.5°C	NM_001243427.1
	R- CCTCTTCTGCTTGTCCCAAC			
Bovine *ACAT2*	F- CGGATTCTTGTCACCCTGTT	223	61°C	NM_001075549.1
	R- TTTCCACCCTCACTTTGGTC			
Pig *GAPDH*	F- AGAAGGTGGTGAAGCAGG	170	61°C	NM_001206359
	R- GTCGTACCAGGAAATGAGC			
Bovine *GAPDH*	F- CCACCCAGAAGACTGTGGAT	127	61°C	NM_001034034.1
	R- TTGAGCTCAGGGATGACCTT			

To evaluate the relative expressions of *ACAA2* and *ACAT2* genes quantitatively in *Sus scrofa* and *B taurus*, real-time qRT-PCR was conducted using an Applied Biosystems, Step-One Real Time PCR system. The dye EvaGreen (Biotium, USA) was used to determine the quantity of transcript of target genes present in each sample. Each individual sample was quantified in triplicate under the following amplification conditions: 95°C for 10 min initially, and then 40 cycles of 95°C for 15 sec and 60°C for 1 min. Standard curve methods were used to define the efficiency of real-time PCR. The efficiency of amplification of the target gene was compared with that of the endogenous *GAPDH* control transcript [17]. Quantification of mRNA levels was performed using the comparative C_T_ method. The results are reported as the relative expression normalised using the level of the transcript of the endogenous reference [18]–[19].

#### Western blotting analysis

Sixty micrograms of protein extract was diluted 1∶1 with 2× loading buffer (4% sodium dodecyl sulfate (SDS), 20% glycerol, 0.004% bromophenol blue, 25% 0.5 M Tris, and 5% β-mercaptoethanol). Protein extracts were denatured by boiling for 5 min before loading onto a 12% SDS-PAGE gel. Proteins were transferred to nitrocellulose membrane after electrophoresis. The membrane was blocked for two hours at room temperature and incubated with primary and secondary antibodies specific for ACAA2, ACAT2, and β-actin, as listed in Table 2.

**Table 2:** List of primary and secondary antibodies used in western blotting.

Gene	Primary antibody*	Secondary antibody*
*ACAA2*	Mouse monoclonal 1∶50 (catalogue no. sc-100847)	Rabbit anti-mouse, 1∶1,000, (catalogue no. sc-358922)
*ACAT2*	Goat polyclonal 1∶50 (catalogue no. sc-30279)	Donkey anti-goat, 1∶1,000 (catalogue no. sc-2020)
*β- actin*	Mouse polyclonal 1∶500 (catalogue no. sc-2025)	Rabbit anti-mouse, 1∶1,000 (catalogue no. sc-358922)

*All antibodies were from Santa Cruz.

The washed membranes were analysed to observe specific chemiluminescent signals using a Luminescent Image Analyzer (LAS-4000 mini) instrument. The results are depicted as the relative band intensities normalised relative to the band intensities of *β-actin* bands for each gene using Image J software (National Institute of Health, Bethesda, Maryland, USA). The means were compared between JNPs and bovines.

### Functional consequences of the coding of nsSNPs of *ACAT2* of *Sus scrofa* using the sorting intolerant from tolerant (SIFT) tool

Studies on SNPs of ACAT2 (*Sus scrofa*) were performed to identify amino acid substitutions which effect metabolic activities and protein conformations. SIFT (available at http://www.blocks.fhcrc.org/sift/SIFT.html) uses sequence homology to predict whether an amino acid substitution affects protein function; if it does, it can be prioritised for further study. In this study, by using SIFT, we submitted SNP queries, selecting those with an intolerance score below a certain threshold for further study. This threshold was fixed at 0.005 or less. SIFT scores were classified as indicating that the SNP would be damaging (0.00–0.05), potentially damaging (0.051–0.10), borderline (0.101–0.20), or tolerable (0.201–1.00) [20].

### Functional annotation of nsSNPs of ACAT2 (*Sus scrofa*) using protein variation effect analyzer (PROVEAN)

PROVEAN is an online software tool that predicts the possible impact of amino acid substitutions on the structural and functional properties of proteins. The amino acid sequences of ACAT2 (*Sus scrofa*) associated with identified SNPs through SIFT were submitted as queries. A delta alignment scoring system was used, where the scores of each supporting sequence were averaged within and across clusters to generate the final PROVEAN score. This computational tool helps to predict the functional effect of variation in amino acids by selecting the common deleterious SNPs through both SIFT and PROVEAN. A protein variant is said to be “deleterious” if the final score is below a certain threshold (default is −2.5) or “neutral” if the score is above this threshold.

### Protein stability prediction using I-Mutant

I-Mutant (2.0) is a support vector machine based tool (http://folding.biofold.org/cgi-bin/i-mutant2.0.cgi). In this tool the FASTA format of protein sequence of ACAT2 was used as an input to predict the effect of a mutation on its stability.

### Homologous modelling and prediction of root mean square deviation (RMSD)

Protein structures were modelled to compare the structural stability of the native and mutant proteins. In the present study, the three-dimensional structure of *ACAT2* from *Sus scrofa* was generated by subjecting the amino acid sequence in the FASTA format to analysis using the SWISS-MODEL expasy and ESyPred3D tools. The obtained structural model that was identical between these two tools was selected and subjected to structural validation as described below.

### Validation of the model

The constructed homologous protein model was further subjected to evaluation of its internal consistency and reliability. A Psi/Phi Ramachandran plot was generated by PROCHECK analysis. The packing quality of the refined structure was investigated using the PROCHECK Quality Control Check. Furthermore, NOMAD–Ref (Normal Mode Analysis, Deformation, and Refinement) was applied for both the native and mutant models to refine the structures to those with lowest energy. The energy-minimised structures were then used for further analysis for the RMSD calculation.

### Identification of the stabilising residues using SRide and the effect of mutation on the protein using Project Hope

The stabilising residues (SRide) tool was used to predict stabilising residues within native and mutant ACAT2 proteins of *Sus scrofa*. A residue is marked as stabilising if it has a high conservation score, high long-range order, high surrounding hydrophobicity, and belongs to a stabilisation centre. The homologous structures of the native and mutant proteins were uploaded as queries. The Project Hope (Have yOur Protein Explained) tool combines the information to give analyses of the effect of a mutation on the protein structure. The protein sequence was used as the input for selection of the mutant variants. The output was given in the form of the structural variation between mutant and wild-type residues.

### Data analysis

The results from the expression analysis by real-time qRT-PCR and western blotting are expressed as the mean ± SEM. The significant differences between the mean expressions of the genes at P<0.05 were analyzed by Tukey's b- test.

## Results

### Functional annotation of *ACAA2* and *ACAT2* using GO

To investigate the functional similarity in the metabolic processes, genes from same protein family i.e., *ACAA2* and *ACAT2* in *Sus scrofa* and *Bos taurus* respectively were selected for the current study. The phylogenetic trees of ACAA2 and ACAT2 were constructed using Treefam. The Treefam showed that in their respective phylogenetic trees the ACAA2 and ACAT2 of *B taurus* and *S scrofa*, respectively are present in same clade.

From the GO annotation, both genes participate in metabolic processes. The CateGOrizer analysis of the *ACAA2* gene from the two species showed fractional differences with respect to their GO categories. On the basis of GO categories it was found that 35.7% and 18.18% of GO functions of *ACAA2* gene in *Bos taurus* and *Sus scrofa*, respectively, are engaged in the regulation of metabolic processes (Table S1). However, the GO function related to lipid metabolism was absent in ACAA2 of *S scrofa*. Surprisingly, the experimental evidence supported by GO annotations indicated that *ACAT2* from *Sus scrofa* has functional similarity to *ACAA2* and *ACAT2* from *Bos taurus* in terms of involvement of both genes in metabolic processes. Furthermore, the CateGOrizer analysis indicated that equal fractions (75%) of *ACAT2* are involved in the metabolic processes in the two species (Table S1). In addition to functional similarities, expression profiling was also conducted to compare the level of expression within and between the species.

### Validation of mRNA and protein expressions of *ACAA2* and *ACAT2* in the liver tissue of *Sus scrofa* and *Bos taurus*

**Figure 1:**
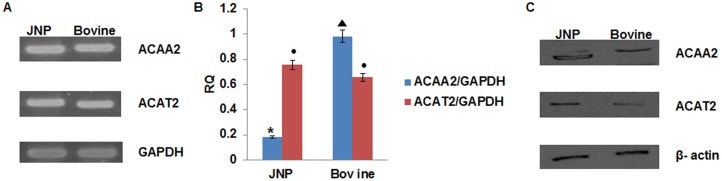
Expression analysis of mRNA and protein levels of *ACAA2* and *ACAT2* in swine and bovine species. a) Expression of mRNA after RT-PCR using 1% agarose gel. b) Relative quantitative expression of *ACAA2* and *ACAT2* genes; bars with different superscripts show significantly different expression in the two species (P<0.05). c) Representative blot expressions of ACAA2, ACAT2, and β-actin proteins obtained by western blotting.

Both RT-PCR and real-time qRT-PCR were performed to clarify the qualitative and quantitative expressions of the genes under study. Specifically, relative mRNA expression levels of *ACAA2* and *ACAT2* were determined after their normalisation relative to the transcript levels of an endogenous reference, *GAPDH*. Here, *ACAA2* exhibited significantly (P<0.05) higher expression in bovine liver than was observed in JNP (Figure 1) although opposite was true for *ACAT2*. It has also been observed that the levels of expression of *ACAT2* are not significantly different in the two species.

The results of the protein expression for the candidate proteins under study were obtained by western blot and are presented as relative band intensities. These intensities of the candidate proteins have been normalised to the area of intensities of β-actin bands. The results from western blot analysis are in line with the results from the relative quantification of mRNA expression levels. Whereas ACAA2 protein was expressed at a significantly (P<0.05) higher level in bovine liver than in JNP (Figure 1), but vice versa expression was observed for ACAT2.

### Retrieval of SNPs and prediction of deleterious SNPs

The present study was carried out to investigate the nsSNPs in the *ACAT2* gene of *Sus scrofa*. Five missense variants, two splice variants, and 14 synonymous SNPs were identified [21]. For further analysis, only the five nsSNPs were selected. The consequences of an amino acid substitution on protein function were estimated using the SIFT tool. It was found that the two nsSNPs, rs321479200 and rs342236888 of ACAT2 were categorised as borderline (0.101–0.20) and tolerable (0.201–1.00) on the basis of their index score i.e. 0.15 and 0.35 respectively (Table 3). In terms of the nucleotide variations, out of nsSNPs, one nsSNP involved a T>C change, another involved a T>G change, and three nsSNPs involved C>T changes.

**Table 3:** List of the deleterious nsSNPs with their prediction tools and scores.

rs id	Substitution native	Allele change	SIFT	PROVEAN	I-mutant (DDG*)
rs342236888	I122V	T/C	0.35	−3.587	−1.89
rs324893294	R139H	C/T	1	5.246	−1.14
rs341690005	A150T	C/T	0.42	0.609	−0.79
rs321479200	R281H	C/T	0.15	−4.012	−1.31
rs334695344	E343D	T/G	0.6	0.673	−0.53

*kcal/mol.

To predict any deleterious effects, the five nsSNPs were furthermore submitted to PROVEAN. On the basis of their scores (−3.587 and −4.012, respectively), the nsSNPs, rs342236888 and rs321479200 of ACAT2 were predicted to be deleterious (Table 3). Analysis of the structural stability associated with these mutations was carried out using I-Mutant; their destabilising effects were evaluated by subtracting the unfolded Gibbs free energy value of the native protein from that of the mutated one.

### Quality analysis of the stability of the homologous model

**Figure 2:**
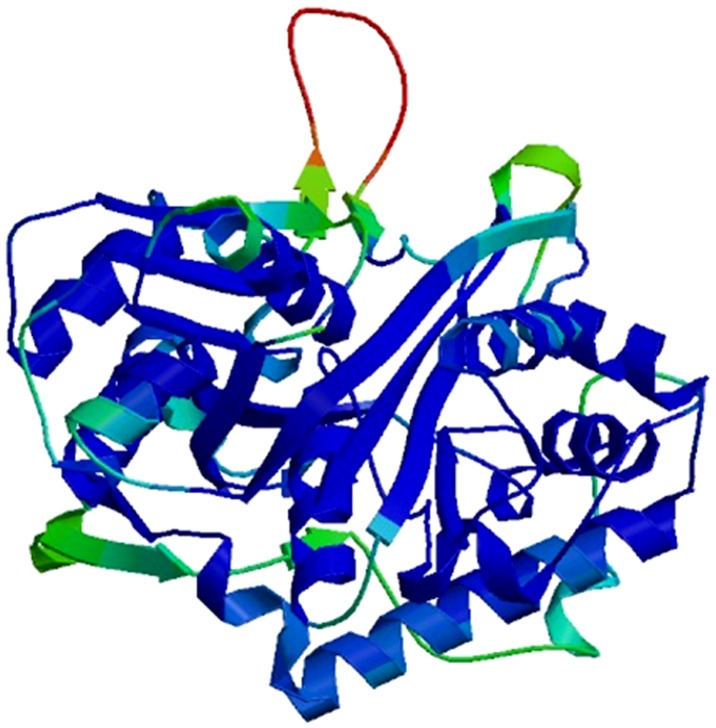
The 3D homologous model of the native ACAT2 protein. A 3D structure of the native ACAT2 protein was prepared using SWISS-MODEL expasy and ESyPred3D. It depicts the native structure in 3D ribbon pattern of chain folding in ACAT2.

**Figure 3:**
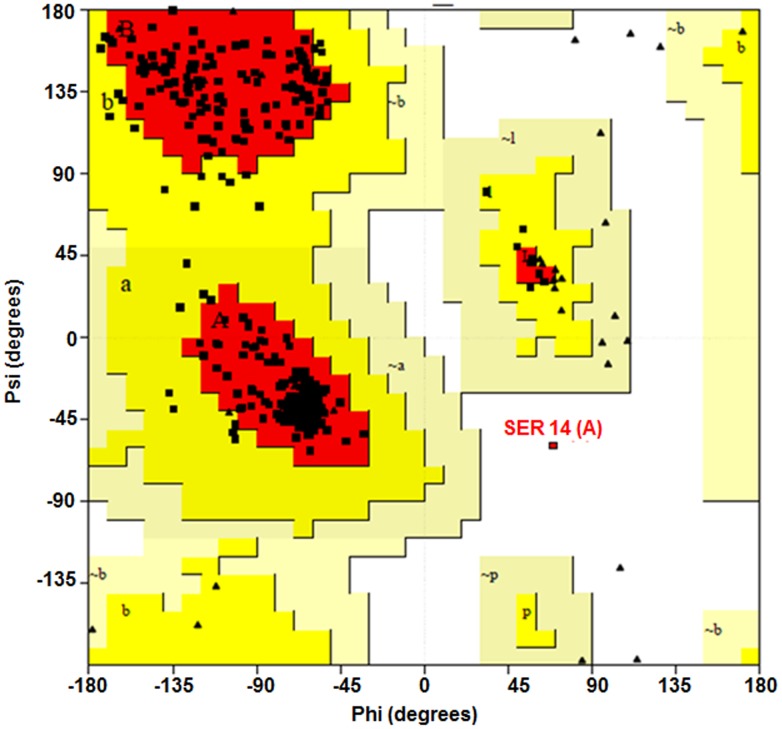
The Ramachandran plot showing the empirical distribution of data points observed in the structure of ACAT2. This plot was used for quality checking and the structural validation of the modelled ACAT2 from *Sus scrofa*. The red, brown, and yellow regions represent the favoured, allowed and generously allowed regions as defined by Procheck.

The 3D structures of the native and mutant proteins were modelled using the SWISS- MODEL expasy and ESyPred3D tools. Both tools gave the same 3D structure using the template PDB ID 1wl 4 of chain A (Figure 2). The validation by PROCHECK verified that 90% of the residues are in the most favoured region in the Ramachandran plot (Figure 3), which indicates the good quality of the model. The QMEAN-Z score (−0.51) for the query model of *ACAT2* indicated the high level of accuracy of the constructed model (Figure S1). In the current study, the total free energy and RMSD values of a nsSNPs i.e. rs342236888 of ACAT2 were found to be −17.531 and 0.82 A°, respectively, whereas those of rs321479200 of ACAT2 were reported to be −16.837 and 2.63 A°. This value of 2.63 A° suggests that rs321479200 is a highly deleterious nsSNP.

**Figure 4:**
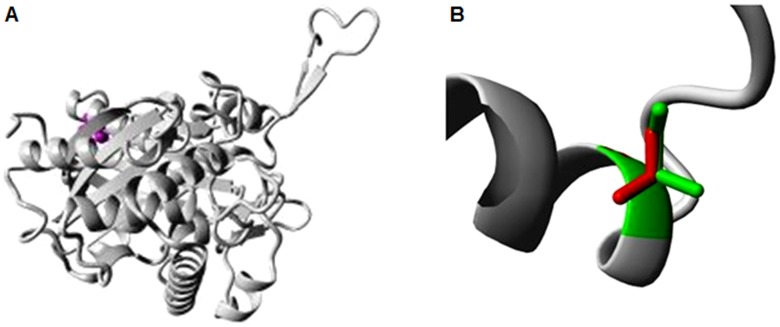
Overview of the ACAT2 protein of *Sus scrofa* showing a mutation of isoleucine to valine (I>V) at position 122. A 3D ribbon pattern was chosen to explain the mutation. a) The protein is coloured grey; the side chain of the residue is shown as small magenta balls. b) Close-up view of the mutation of isoleucine to valine (I>V) at position 122 in ACAT2 of *Sus scrofa*. The side chains of both wild-type and mutant residues are shown in green and red, respectively.

**Figure 5:**
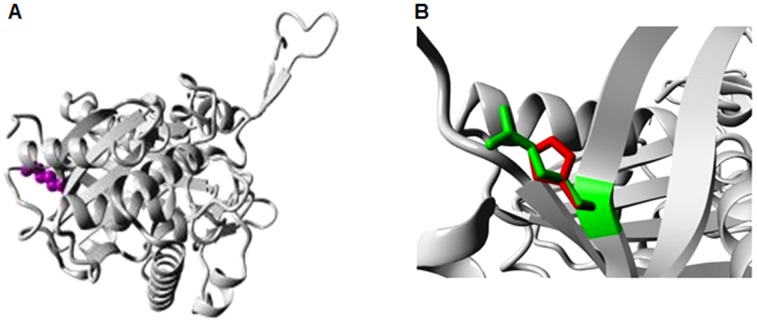
Overview of the ACAT2 protein of *Sus scrofa* showing mutation of arginine to histidine (R>H) at position 281. A 3D ribbon pattern was chosen to explain the mutation. a) The whole protein is presented in grey, with the side chain of the residue as small magenta balls. b) Close-up view of the mutation of arginine to histidine (R>H) at position 281 in ACAT2 of *Sus scrofa*. The side chains of both wild-type and mutant residues are shown in green and red, respectively.

The stabilising residues in the native and mutant protein models were identified using the SRide tool; variations in these stabilising residues are shown in Table 4. The 13 stabilising residues, those in the native protein were compared with their counterparts in the mutants. The stability of the native ACAT2 protein was compared with its mutated homologous structures (Figure 4 and 5) showing the causative mutations i.e. I122V and R281H. It was found that nsSNP-rs321479200 (R281H) was associated with more residues having a stabilising effect, which may be important in terms of structure and function.

**Table 4:** List of stabilising residues between the native and deleterious nsSNPs.

Substitution	Existing residues													Newly formed
**Native**	VAL17	VAL18	ILE19	VAL64	ILE65	VAL124	ALA125	GLY126	ALA269	MET273	LYS274	GLY387	VAL388	----
**I122V**	VAL17	VAL18	ILE19	VAL64	----	VAL124	ALA125	----	ALA269	MET273	LYS274	GLY387	VAL388	Phe57
**R281H**	----	----	ILE19	VAL64	----	VAL124	ALA125	----	ALA269	----	LYS274	----	----	Gly78, Ser232, Gly374

### Effect of mutation on protein function

The possible effects of mutations at specific residues on protein function were also evaluated. Interestingly, both the native residues, namely, isoleucine and arginine, of ACAT2 protein of *Sus scrofa* are part of an interpro domain named thiolase (IPR002155). This domain was classified into the following GO categories: 0008152 (metabolic process), 0016747 (transferring acyl groups other than amino-acyl groups), and 0016740 (transferase activity). These same residues, isoleucine and arginine, are also part of an interpro domain called thiolase-like, subgroup (IPR016038), with the GO categories of 0003824 (catalytic activity) and 0008152 (metabolic process). These residues are also part of a third interpro domain named thiolase, N-terminal (IPR020616), which plays a role in metabolic processes (GO: 0008152), transferase activity, and transferring acyl groups other than amino-acyl groups (GO: 0016747). Therefore, changes of these residues can affect functions of the protein.

The wild-type and mutant amino acids of ACAT2 protein of *Sus scrofa* differ in size. The mutation in a nsSNP-rs342236888 of ACAT2 at position 122, is associated with a change of residue from isoleucine to valine, thus changes the charge (Figure 4). In addition, the mutant residue is smaller than the wild-type one, which leads to empty space in the core of the protein. These differences can disturb the core structure of this important domain and thereby affect the catalytic activity of a nsSNP-rs342236888 of ACAT2.

Similarly, for a nsSNP-rs321479200 of ACAT2 at position 281, mutation of Arg to His alters the charge between the wild-type and mutant amino acids (Figure 5). Specifically, this mutation causes a loss of charge, as well as interactions with molecules different from those that interact with the wild type. The difference in size between the mutant and the wild type would also possibly cause a loss of external interactions. The residue is located on the surface of ACAT2 and is not found to be in contact with other domains with known functions. However, contacts with other molecules or domains are possible and might be affected by this mutation.

## Discussion

To our knowledge, this study is the first attempt to characterise nsSNPs that are related to the metabolism and expression of *ACAT2* in pig liver. Results of this study show that nsSNPs are considered to be potentially important for the functional and structural analysis of proteins. Such nsSNPs are of particular interest, given that they might provide markers of selection. These 5 nsSNPs were functionally annotated and were subjected to analysis using the SIFT and PROVEAN tools to predict their potential deleterious effects. The tolerance index of selected deleterious nsSNPs is inversely related to the functional impact of the corresponding amino acid residues [22]–[23]. rs342236888 (I122V) and rs321479200 (R281H) were selected as deleterious nsSNPs in this study (low tolerance indexes of −1.89 and −1.31, respectively). The *in silico* analysis indicated that misfolding and intermolecular interactions of the *ACAT2* gene, can have marked effects on the structure and functions of the protein. Thiolase domain has been enlisted as an important in the key enzymatic pathways such as fatty acid, steroid and polypeptide synthesis. It has also been reported to play a role in transferase activity and metabolic process. Therefore, it can be predicted that due to the mutations, I122V and R281H, nsSNPs might have a direct influence on the metabolical functions of ACAT2 in *S scarofa* [24].

Moreover, the food industry has been criticized in particular for the decline in the quality of pork from lean breeds [25]. Further, it has been observed that the properties of lean meat are greatly influenced by its chemical composition. The amino acid contents of semimembranosus muscle (threonine, isoleucine, lysine, aspartic acid, serine, proline) increase with an increase in the proportion of lean meat [26]. Moreover, in our study where isoleucine mutates to valine will result in the decrease in the number of residues of isoleucine in the native form of ACAT2 of *Sus scrofa*. Therefore, it can be one of the contributing factors for decrease in the amount of lean meat.

GO categories have been used previously to characterise protein function and to elucidate trends in protein datasets [27]. GO terms here helped to identify biological processes that could be affected by the mutations. Our finding that *ACAT2* is mainly responsible for metabolic processes involving lipid metabolism (Table S1) is supported by previous study [28]. It has been well established that both genes are involved in metabolic pathways [29] and belong to the same protein family. It has also been observed that ACAT2 is mostly involved in lipid metabolism; in the present investigation, it has shown more metabolic activity than ACAA2. Validation in the laboratory reported empirical evidence of actual biological similarities between ACAT2 and ACAA2. Later, this concept was supported by the similarities during GO annotation. Significantly higher expression of *ACAT2* in the JNP liver supports the metabolic role of *ACAT2* and its importance in physiological processes. In an earlier study, higher expression of *ACAT2* was also linked to cholesterol levels, which are in turn reported to be linked to increased lean mass [8].

ACAT2 plays an important role in efficient cholesterol absorption, lipoprotein metabolism, and atherosclerosis [28], [30]–[31]. Cholesterol plays a direct role in inflammation. It is also believed that inflammation plays a significant role in muscle building [8]. Moreover, an earlier study has also reported significant role of cholesterol in the muscle building under the dose dependent manner [32]. Therefore, it has been concluded that cholesterol levels are dose-dependently associated with increased muscle size and strength.

ACAT2 can esterify cholesterol and oxysterols in humans and other animals [33]–[34]. Cholesterol is also essential for the formation of lipid rafts, which function in the assembly of the components of signalling pathways through sorting of proteins and construction of signalling complexes [35]. Earlier studies have reported that these signalling pathways contribute to the development of skeletal muscle [36]–[39]. Therefore, significantly high expression of *ACAT2* in *S scrofa* during the current study indicates that it can also be involved in the high muscle growth.

In conclusion, this study demonstrates the significant application of bioinformatic tools to clarify the changes in functions associated with the metabolic processes in which *ACAT2* participates. *In silico* analysis of the preferred nsSNPs of the *ACAT2* gene that are most strongly related to metabolic activity could explain the alteration in the metabolic activity caused by the mutations. Annotation of the effects of “I to V” substitution at residue 122 and “R to H” at residue 281 of ACAT2 might be useful as selection marker for molecular diagnosis. In terms of the importance of this loss of arginine (R), previous researchers suggested its role for improving meat quality [40]. Further evidence also indicates that arginine regulates the partitioning of dietary energy in favour of muscle protein accretion and fat reduction in animals [41]. Moreover, it was found that activation of endogenous arginine synthesis increased the average daily weight gain in piglets, compared with that in a control group [40], [42].

Furthermore, our GO analysis and evaluation of mRNA and protein expression in the laboratory indicates that *ACAT2* plays more important role in lipid and cholesterol metabolism in pig than bovine. Therefore, this study can be used as a stepping stone for planning future studies on the role of nsSNPs in metabolic processes, especially in pigs. The associations of SNPs in *ACAT2* with fertility and production traits can be used to plan studies for the improvement of growth performance and fertility traits in Jeju native pigs.

## Supporting Information

Figure S1 **QMEAN-Z score of the model for** ***ACAT2*** **of** ***Sus scrofa*** **.** The red legend indicates the QMEAN-Z score.
(TIF)

Table S1 **Gene ontology analysis suggests the biological processes associated with functioning of the** ***ACAA2*** **and** ***ACAT2*** **genes in** ***Sus scrofa*** **and** ***Bos taurus.***
(DOCX)
