# Supplementary figures and images for: An Approach to Identify SNPs in the Gene Encoding Acetyl-CoA Acetyltransferase-2 (ACAT-2) and Their Proposed Role in Metabolic Processes in Pig
Source: PLoS One. 2014 Jul 22;9(7):e102432. doi: 10.1371/journal.pone.0102432 (PMC4106792; doi:10.1371/journal.pone.0102432)

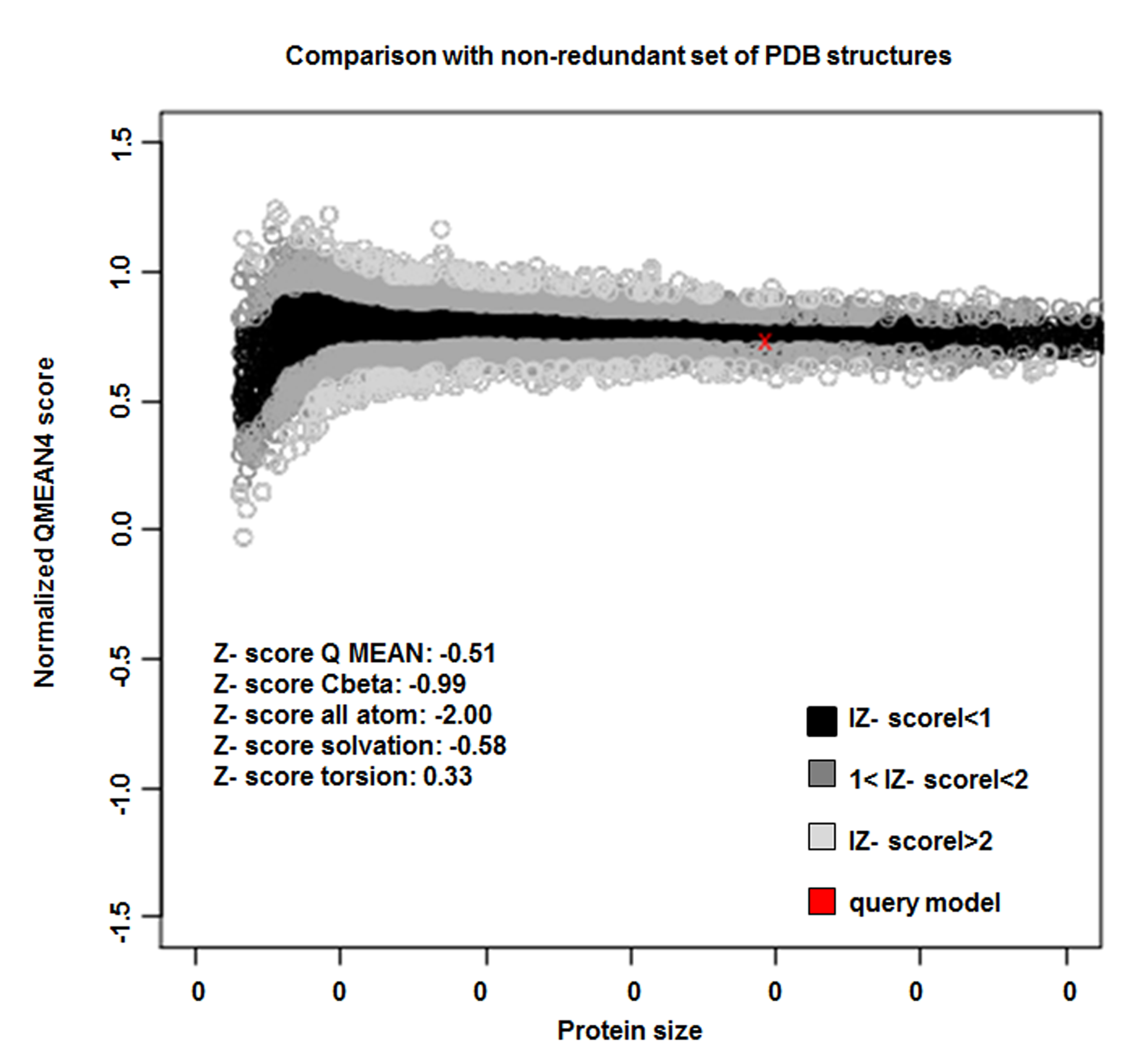

Supplement: Figure S1 — QMEAN-Z score of the model for ACAT2 of Sus scrofa . The red legend indicates the QMEAN-Z score. (TIF) [file pone.0102432.s001.tif]
