# Supplementary material for: An Approach to Identify SNPs in the Gene Encoding Acetyl-CoA Acetyltransferase-2 (ACAT-2) and Their Proposed Role in Metabolic Processes in Pig
Source: PLoS One. 2014 Jul 22;9(7):e102432. doi: 10.1371/journal.pone.0102432 (PMC4106792; doi:10.1371/journal.pone.0102432)
Supplement: Table S1 — Gene ontology analysis suggests the biological processes associated with functioning of the ACAA2 and ACAT2 genes in Sus scrofa and Bos taurus. (DOCX) [file pone.0102432.s002.docx]

**An approach to identify SNPs in the gene encoding acetyl-CoA acetyltransferase-2**

**(*ACAT*-*2*) and their proposed role in metabolic processes in pig**

Simrinder Singh Sodhi^1+^, Mrinmoy Ghosh^1+^, Ki-Duk Song^2^, Neelesh Sharma^1^, Jeong Hyun Kim^1^, Nameun Kim^1^, Sung Jin Lee^3^, Chulwoong Kang^4^, Sung Jong Oh^1^ and Dong Kee Jeong^1,5^*

^1^Department of Animal Biotechnology, Faculty of Biotechnology, Jeju National University, Jeju-si, Jeju-do, South Korea

^2^The Animal Genomics and Breeding Center, Hankyong National University, Anseong-si, Gyeonggi-do, South Korea

^3^Department of Animal Biotechnology, College of Animal Bioscience and Technology, Kangwon National University, Chuncheon, South Korea

^4^Department of Mechanical and System Engineering, College of Engineering, Jeju National University, Jeju-si, Jeju-do, South Korea

^5^Sustainable Agriculture Research Institute (SARI), Jeju National University, Jeju-si, Jeju-do, South Korea

+ Authors have contributed equally

*Corresponding author:

Prof. Dong Kee Jeong,

Department of Animal Biotechnology,

Faculty of Biotechnology, Jeju National University,

Ara-1 Dong, Jeju-Si, Jeju-Do 690-756, South Korea.

Mobile: +82-10-8713-8045, Fax: +82-64-725-2403-3331

Email: [dkjeong@jejunu.ac.kr](mailto:dkjeong@jejunu.ac.kr)

| **SUPPLEMENTARY INFORMATION** |
| --- |

1. **Table S1……………………………..……………..……p.3**

| Table S1: Gene ontology analysis suggests the biological processes associated with functioning of the *ACAA2* and *ACAT2* genes in *Sus scrofa* and *Bos taurus.* | | | | | |
| --- | --- | --- | --- | --- | --- |
|  | | | | | |
| GO Class ID* | **Definitions** | **Counts**** | | **Fractions***** | |
|  |  | ***Sus Scrofa*** | ***Bos taurus*** | ***Sus Scrofa*** | ***Bos taurus*** |
| *ACAA2* | | | | | |
| GO:0006915 | apoptosis | 4 | 4 | 36.36% | 28.57% |
| GO:0008152 | metabolism | 2 | 3 | 18.18% | 21.43% |
| GO:0006629 | lipid metabolism | NA | 2 | NA | 14.29% |
| GO:0016265 | death | 2 | 2 | 18.18% | 14.29% |
| GO:0005739 | mitochondrion | 2 | 2 | 18.18% | 14.29% |
| GO:0042981 | regulation of apoptosis | 1 | 1 | 9.09% | 7.14% |
| *ACAT2* | | | | | |
| GO:0008152 | metabolism | 2 | 2 | 50.00% | 50.00% |
| GO:0005739 | mitochondrion | 1 | NA | 25.00% | NA |
| GO:0006629 | lipid metabolism | 1 | 1 | 25.00% | 25.00% |
| GO:0009058 | biosynthesis | NA | 1 | NA | 25.00% |
|  | | | | | |

*** Go terms/ class IDs:** GO terms into each ancestral term are independent of one another. The counted results may be selectively used and percentages recalculated as the selected terms represent a well-covered spectrum of the scope which helps to avoid redundancy and may make best sense for the data set.

**** Counts :** Number of similar GOs with the current GO

***** Fractions :** Share of biological process shown with respect to GO functions
